# Trouble psychotique partagé atypique: à propos d’une observation

**DOI:** 10.11604/pamj.2017.27.12.9481

**Published:** 2017-05-05

**Authors:** Marcellin Bugeme, Papa Alioune Seck, Paul Makinko Ilunga, Olivier Mukuku

**Affiliations:** 1 Service de Neuro-Psychiatrie, Cliniques Universitaires de Lubumbashi, Faculté de Médecine, Université de Lubumbashi, Centre Neuropsychiatrique Dr Joseph Guislain/Frères de la Charité, Lubumbashi, RDCongo; 2 Centre Hospitalier National Universitaire de Fann, Dakar, Sénégal; 3 Département de Pédiatrie, Cliniques Universitaires de Lubumbashi, Faculté de Médecine, Université de Lubumbashi, Lubumbashi, RDCongo

**Keywords:** Folie à deux, folie partagé, atypique, délires induits, délires partagés, délires collectifs, Folie à deux, shared madness, atypical, induced delusions, shared delusions, collective delusions

## Abstract

Le trouble psychotique partagé est une entité psychiatrique considérée comme rare et caractérisée par la transmission des idées délirantes d'un patient dit “inducteur“, à un autre dit “induit“. Nous rapportons une observation clinique de trouble psychotique partagé atypique mettant en jeu un délire de persécution et un autre de grandeur induits chez un adolescent par deux sujets (grand-père et oncle paternel) ayant présenté deux entités psychiatriques différentes, mais tous les partagent à un même sujet (le troisième). La nature du contenu transmis au troisième sujet était la somme des idées des deux premiers. Ces trois sujets vivaient en association proche, dans un milieu clos et coupé de l'extérieur.

## Abstract (en)

Shared psychotic disorder is a rare entity characterized by the transmission of delusional symptoms from one patient (“inducer“) to another (“induced“ partner). We here report a clinical observation of atypical shared psychotic disorder involving delusion of persecution and of grandeur in a teenager induced by two subjects (grand-father and paternal uncle) with two different types of psychiatric disorders, all of which present in the same subject (third-party). The nature of the content transmitted to the third subject was the sum of the ideas of the first two subjects. These three subjects had a close relationship and lived in a closed environment, without access to the outside world.

## Introduction

Le trouble psychotique partagé ou trouble délirant induit est une entité psychiatrique caractérisée par des idées délirantes semblables partagées par deux ou plusieurs personnes qui ont une relation proche. Il a été d'abord décrit par Jules Baillarger en 1860, qui a nommé cette condition comme “folie communiquée“. Il a été différemment appelé : psychose d'association, trouble paranoïde partagé, la folie contagieuse. C'est en 1877 que Lasègue et Falret ont pu inventer le terme “folie à deux“ ou “psychose de deux“ [1, 2]. C'est une entité psychiatrique considérée comme rare ; aucune statistique n'est disponible sur son incidence et sa prévalence. Les données de la littérature disponibles sont principalement basées sur la description de cas cliniques. Trois enquêtes ayant passé en revue de toutes les présentations de cas cliniques sur cette pathologie indiquent que 242 cas ont été publiés de 1877 à 2005 [3–5]. Cette entité implique souvent deux personnes mais des rares cas où plus de deux personnes sont impliquées ont été décrits et porte respectivement les noms de folie à deux, folie à trois, folie à quatre ou folie à famille, selon le nombre de personnes impliquées [6, 7]. Partant du principe d'une étroite relation préexistant entre les deux individus malades, le couple délirant est variable ; il peut s'agir des conjoints, des personnes issues d'une même famille, des voisins, voire même des amis. L'observation clinique que nous rapportons décrit des idées délirantes partagées chez un adolescent par deux de ses parents (grand-père et oncle paternels) avec lesquels il vivait en étroite relation. Dans cette observation clinique, l'atypie consiste en la mise en jeu des idées délirantes différentes (de grandeur et de persécution) chez deux sujets inducteurs avec un autre sujet considéré comme induit.

## Patient et observation

Il s'agit d'une observation clinique impliquant trois sujets (A, B et C). Le sujet A veuf et âgé 69 ans était grand-père paternel de du sujet C. Le sujet B divorcé et âgé de 51 ans était fils du sujet A et oncle paternel du sujet C. Le sujet C âgé de 14 ans, célibataire, était petit-fils du sujet A et neveu du sujet B. Ils avaient respectivement 12, 15 et huit années d'études. Les trois sujets vivaient seuls dans une maison et chacun dans une chambre. Ils étaient isolés du reste de la famille depuis plusieurs années. Dans leurs antécédents personnels médico-psychiatriques, nous avons noté une démence présénile chez le sujet A et une encéphalopathie hépatique au stade III secondaire à un hépato carcinome chez le sujet B. Le sujet s'était quant à lui sans antécédent pathologique particulier.

Le début des troubles du sujet C remonterait à environ une semaine marqué par la survenue d'une agitation psychomotrice. En effet il s'occupait des sujets A et B. Ils vivaient grâce à l'aide de quelques membres de leur famille, animés de bonne foi, qui contribuaient à leur ravitaillement. Le sujet C qui partait à chaque fois récupérer le ravitaillement avait arrêté de retirer cette aide. C'est par la suite que les membres de la famille, inquiets de la situation, décidaient de s'enquérir de la situation. Ils avaient ainsi constaté que le sujet C présentait une instabilité psychomotrice motivant sa consultation en pédiatrie puis son transfert au service de psychiatrie pour une meilleure prise en charge. Au terme de l'anamnèse et au su d'une dynamique familiale perturbée, une visite à domicile a été effectuée pour s'enquérir de l'état de santé des sujets A et B.

A l'observation psychiatrique, nous avons noté: 1) Le sujet C présentait un syndrome délirant aigu à début rapidement progressif, à thématique de persécution, hypocondriaque et de grandeur: il disait *« mon entourage veut ma mort car je suis le président de la république et je suis bourré d'argent… »* il disait également: *«j'ai des douleurs abdominale au niveau de côté droit et je me vois vomir du sang.»* à mécanisme imaginatif, flou et mal systématisé avec une forte adhésion et comme réaction une hyper religiosité avec des séances de prière excessive; 2) Le sujet A avait un syndrome démentiel avec une agitation physique puérile et verbale avec logorrhée et une insomnie. Il exprimait des idées délirantes de grandeur : il disait *« je suis le président de la république et je suis bourré d'argent ».* Il présentait une amnésie de fixation et d'évocation et aussi une désorientation temporo-spatiale; 3) Le sujet B présentait une asthénie, une douleur de l'hypocondre droit avec hépatomégalie, un ictère conjonctival bilatéral, une hématémèse avec parfois des vomissements incoercibles. Il présentait une difficulté de marcher, une désorientation temporo-spatiale et une insomnie. Il exprimait également des idées délirantes de persécution à mécanisme essentiellement imaginatif évoluant depuis deux mois.

Le sujet C était hospitalisé et traité avec de la Chlorpromazine (50 mg deux fois par jour pendant trois jours), de l'Halopéridol (cinq mg deux fois par jour pendant deux semaines) et de Valproate de Sodium (500 mg deux fois par jour pendant deux semaines). Il avait bénéficié également d'une psychothérapie de soutien et au bout de deux semaines, son état clinique s'était stabilisé. Après son hospitalisation, il a été réinséré dans un autre milieu social loin des sujets A et B.

## Discussion

Les premières descriptions de cette entité furent apportées par Lasègue et Falret en 1877: deux sujets, vivant en association proche, dans un milieu clos et isolé, partagent des idées délirantes sur le même thème. Le délire est imposé à un individu crédule, faible d'esprit ou suggestible et ayant peu d'accès à la critique: c'est le cas princeps de la « folie à deux » [1].

Le trouble psychotique partagé est caractérisé par les idées délirantes semblables partagées par des personnes qui ont une relation étroite et vivent typiquement ensemble dans l'isolement social relatif [7, 8]. Dans notre observation, les trois individus restaient ensemble et avaient eu un contact minimal avec des parents ou des amis. Les sujets A et B (oncle et grand-père paternels du sujet C) étaient dominants. Ils étaient clairement ceux qui avaient initié leurs délires qu'ils avaient progressivement imposés sur le sujet A qui, de nature, était passif, dépendant et soumis. Selon Kumar [8], l'inducteur et l'induit vivent dans un isolement croissant et ont tendance à devenir de plus en plus méfiants. Ils se sentent menacés par une atmosphère apparemment de plus en plus hostile qui, à son tour, conduit à la production de la réaction paranoïaque et par la suite une psychose paranoïaque.

L'étude de Lasègue et Falret [1] relève trois conditions régissant le trouble psychotique partagé. Premièrement, il s'agit de la loi de la différence intellectuelle et de caractère. Deuxièmement, la loi du milieu clos implique que les individus partagent absolument tout, en dehors de la moindre influence du monde extérieur. Et troisièmement, la loi de la vraisemblance selon laquelle le délire doit être vraisemblable, de manière à ce que la conviction délirante puisse être communiquée entre les différents individus. Notre observation répond également aux directives pour le diagnostic de trouble délirant induit selon la CIM-10 [9] et aux critères diagnostiques pour un trouble psychotique partagé selon le DSM-IV [10]. Mais une particularité est à noter dans celle-ci: la présence de deux sujets inducteurs et d'un seul sujet induit. L'aspect clé dans l'étiologie et la psychopathologie de folie partagée est la nature de la relation entre l'inducteur et l'induit [8]. Dans notre observation, le sujet C était intimement lié à ses deux parents (sujets A et B), à la fois dans la proximité physique et dans l'intensité émotionnelle. Le sujet C (induit) avait partagé les idées délirantes de grandeur du sujet A (inducteur 1) et celles de persécution du sujet B (inducteur 2) dans l'intégralité. Plusieurs auteurs ont insisté sur la fonction du délire pour l'un ou l'autre des sujets : le délire semble par certains égards apparaitre profitable à l'un et l'autre des protagonistes [11], il permet la conservation de l'isolement et la cohésion du groupe et le sujet secondaire pourrait accepter le délire pour maintenir la relation à son codélirant [12]. Parfois l'éclosion du délire chez le sujet primaire est favorisée par le sujet secondaire, expliquant ainsi en retour la plus grande facilité de ce dernier à adhérer au délire [13].

La principale mesure thérapeutique préconisée par les aliénistes qui ont initialement décrit ce tableau clinique, consiste à séparer les patients. Le cas secondaire renonce alors assez rapidement mais parfois très lentement à cette croyance d'allure délirante. Il retrouve dès lors son assise symbolique antérieure tandis que le cas primaire maintient intacte sa conviction [1, 10]. Dans le cas que nous rapportons le sujet A avait été hospitalisé et séparé des sujets B et C. Mais il faut noter qu'il n'est pas toujours évident que la seule séparation puisse permettre le rétablissement du sujet induit. C'est pourquoi, comme le suggèrent d'autres auteurs [6, 8, 14], nous y avons aussi adjoint un traitement psychopharmacologique pour stopper son état d'agitation et réguler son humeur.

## Conclusion

Dans ce cas rapporté, les inducteurs qui avaient partagé leurs idées délirantes avaient une position familiale très forte et étaient plus âgés et plus instruits que le sujet induit ou secondaire qui, lui, avait une personnalité dépendante. C'est un cas rare et c'est le premier que nous ayons pu observer dans notre milieu et dans la littérature où l'on retrouve deux inducteurs et un induit. La folie à deux est une entité nosographique qui semble encore difficile à cerner ne serait-ce que sa sémiologie ou son étiologie.

## Conflits d’intérêts

Les auteurs ne déclarent aucun conflit d'intérêts.
